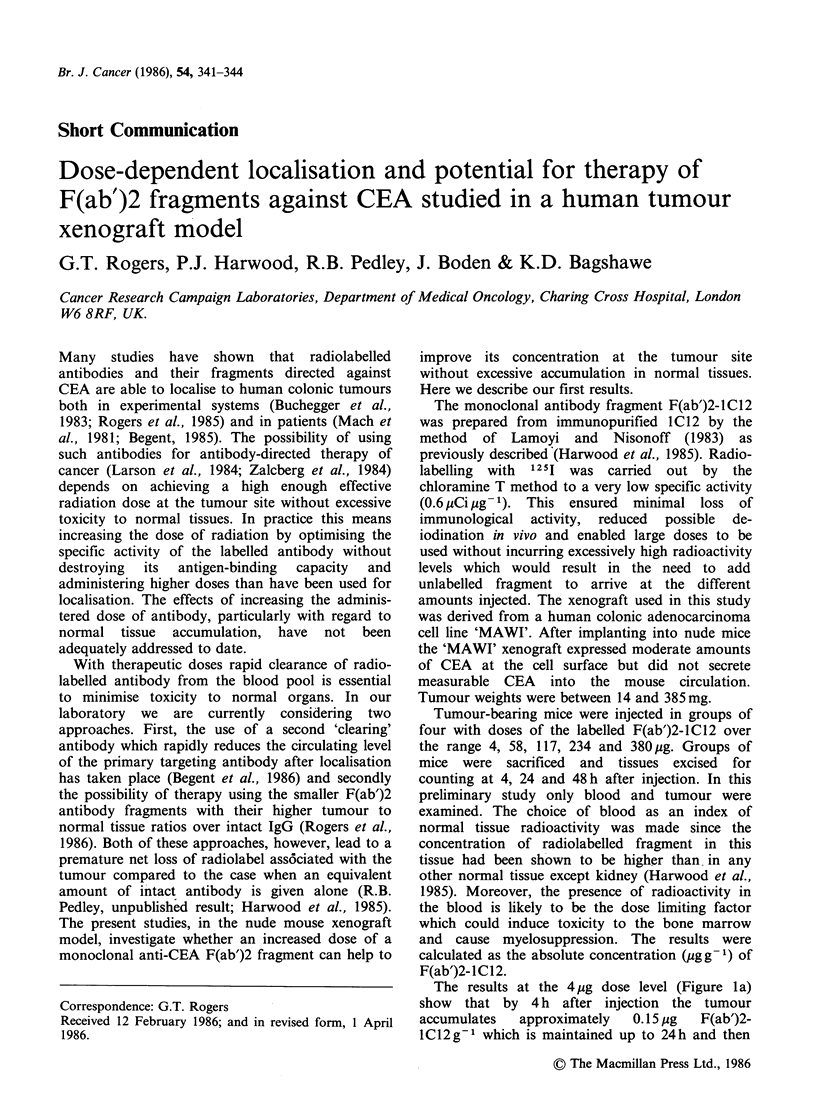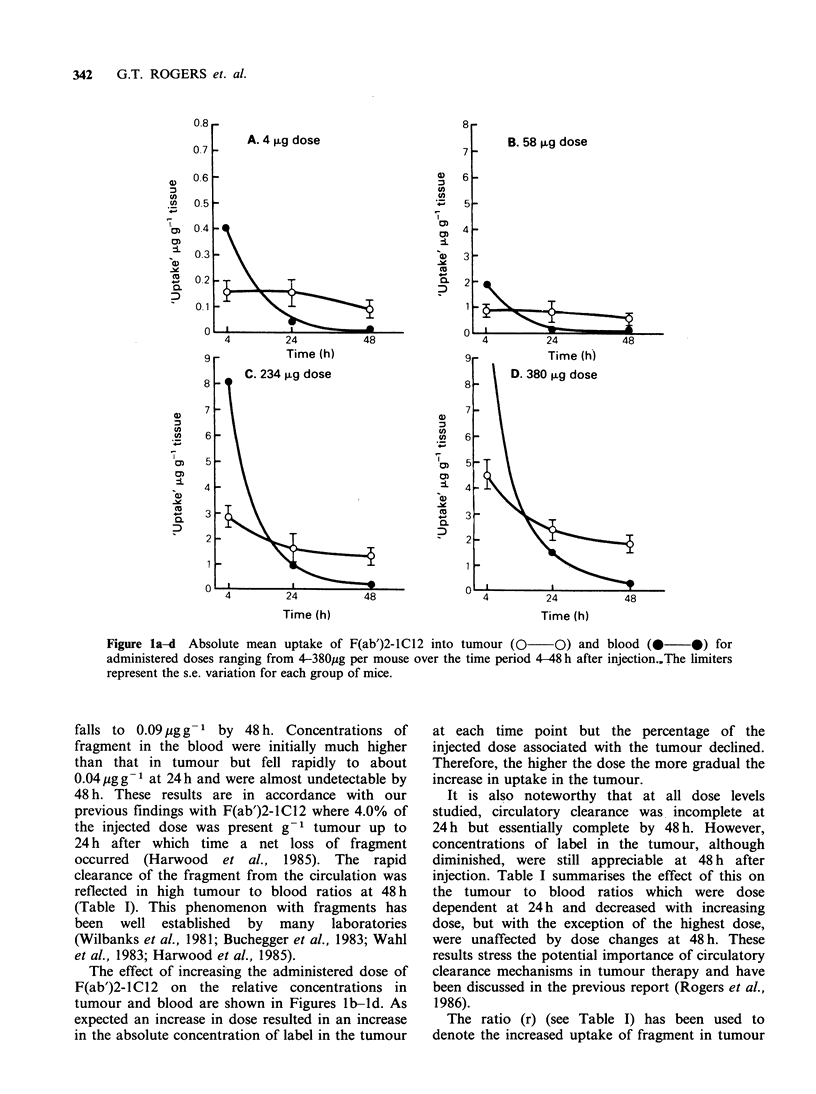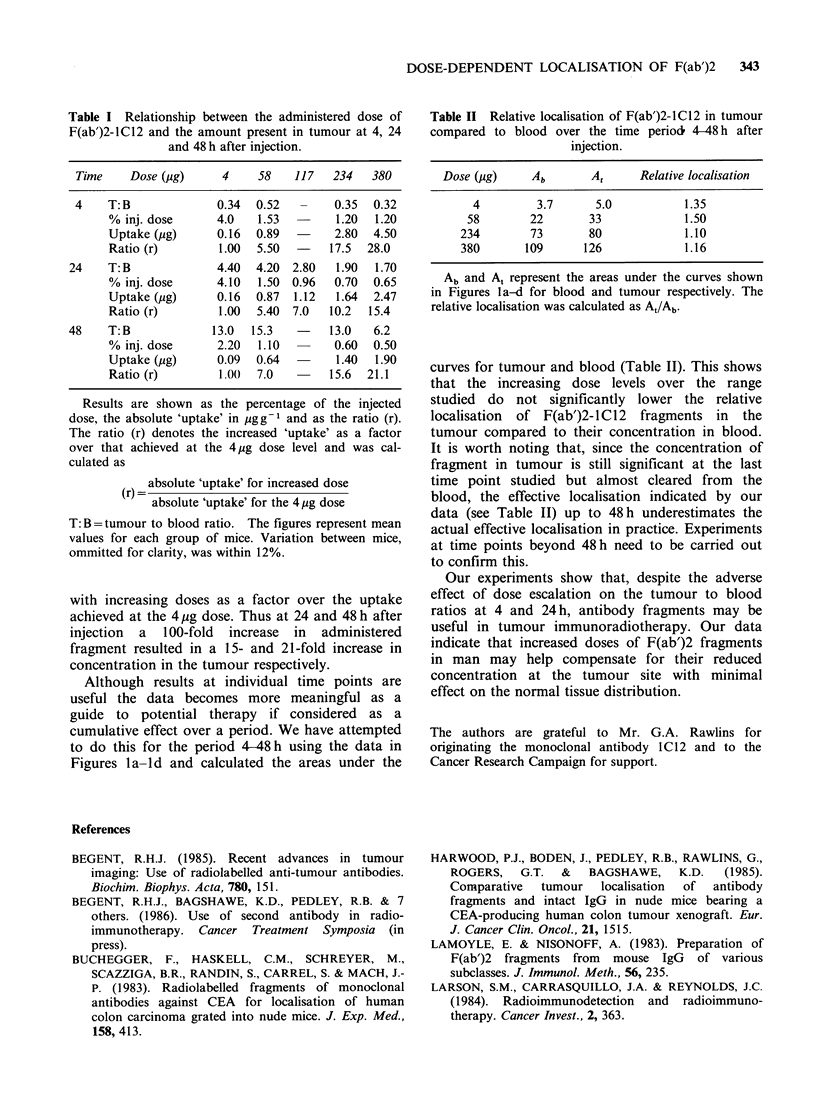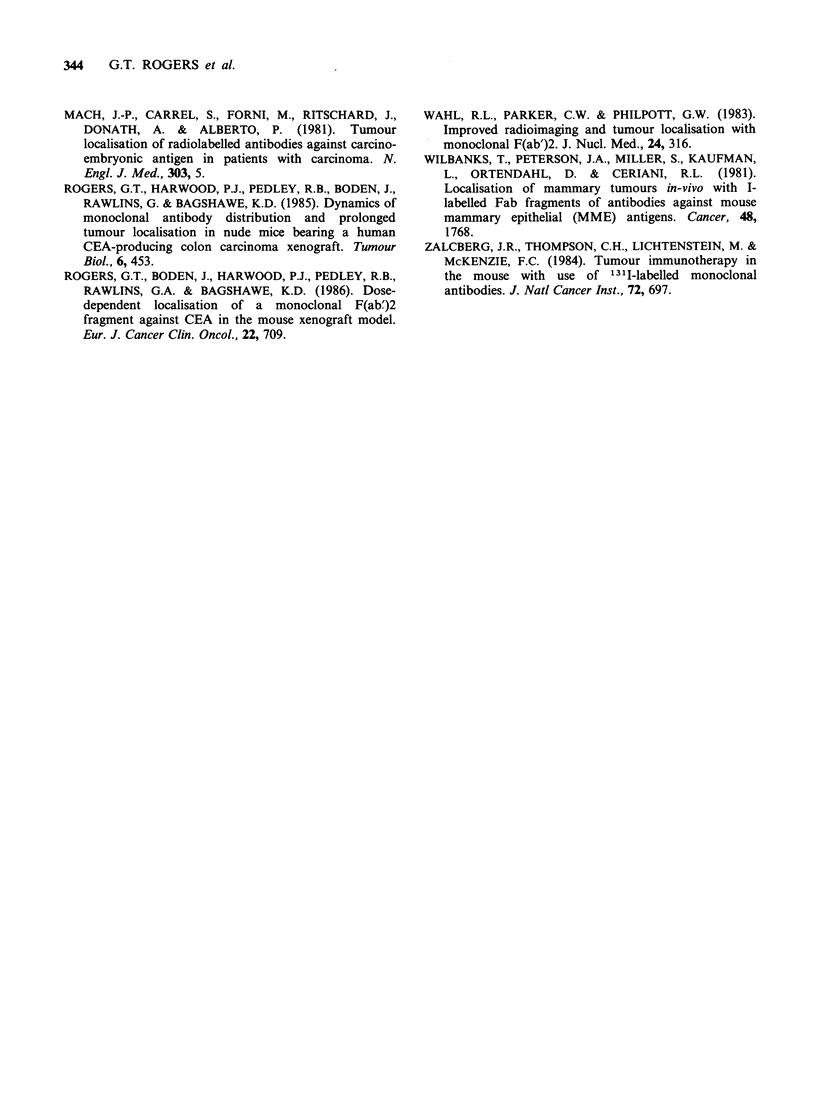# Dose-dependent localisation and potential for therapy of F(ab')2 fragments against CEA studied in a human tumour xenograft model.

**DOI:** 10.1038/bjc.1986.183

**Published:** 1986-08

**Authors:** G. T. Rogers, P. J. Harwood, R. B. Pedley, J. Boden, K. D. Bagshawe


*Br. J. Cancer* (1986), **54**, 341–344

### Short Communication

### Dose-dependent localisation and potential for therapy of F(ab′)2 fragments against CEA studied in a human tumour xenograft model

G.T. Rogers, P.J. Harwood, R.B. Pedley, J. Boden & K.D. Bagshawe

*Cancer Research Campaign Laboratories, Department of Medical Oncology, Charing Cross Hospital, London W6 8RF, UK.*

Many studies have shown that radiolabelled antibodies and their fragments directed against CEA are able to localise to human colonic tumours both in experimental systems (Buchegger *et al.*, 1983; Rogers *et al.*, 1985) and in patients (Mach *et al.*, 1981; Begent, 1985). The possibility of using such antibodies for antibody-directed therapy of cancer (Larson *et al.*, 1984; Zalcberg *et al.*, 1984) depends on achieving a high enough effective radiation dose at the tumour site without excessive toxicity to normal tissues. In practice this means increasing the dose of radiation by optimising the specific activity of the labelled antibody without destroying its antigen-binding capacity and administering higher doses than have been used for localisation. The effects of increasing the administered dose of antibody, particularly with regard to normal tissue accumulation, have not been adequately addressed to date.

With therapeutic doses rapid clearance of radiolabelled antibody from the blood pool is essential to minimise toxicity to normal organs. In our laboratory we are currently considering two approaches. First, the use of a second 'clearing' antibody which rapidly reduces the circulating level of the primary targeting antibody after localisation has taken place (Begent *et al.*, 1986) and secondly the possibility of therapy using the smaller F(ab′)2 antibody fragments with their higher tumour to normal tissue ratios over intact IgG (Rogers *et al.*, 1986). Both of these approaches, however, lead to a premature net loss of radiolabel associated with the tumour compared to the case when an equivalent amount of intact antibody is given alone (R.B. Pedley, unpublished result; Harwood *et al.*, 1985). The present studies, in the nude mouse xenograft model, investigate whether an increased dose of a monoclonal anti-CEA F(ab′)2 fragment can help to improve its concentration at the tumour site without excessive accumulation in normal tissues. Here we describe our first results.

The monoclonal antibody fragment F(ab′)2-1C12 was prepared from immunopurified 1C12 by the method of Lamoyi and Nisonoff (1983) as previously described (Harwood *et al.*, 1985). Radiolabelling with $^{125}$I was carried out by the chloramine T method to a very low specific activity (0.6 $\mu$Ci $\mu g^{-1}$). This ensured minimal loss of immunological activity, reduced possible deiodination *in vivo* and enabled large doses to be used without incurring excessively high radioactivity levels which would result in the need to add unlabelled fragment to arrive at the different amounts injected. The xenograft used in this study was derived from a human colonic adenocarcinoma cell line 'MAWI'. After implanting into nude mice the 'MAWI' xenograft expressed moderate amounts of CEA at the cell surface but did not secrete measurable CEA into the mouse circulation. Tumour weights were between 14 and 385 mg.

Tumour-bearing mice were injected in groups of four with doses of the labelled F(ab′)2-1C12 over the range 4, 58, 117, 234 and 380 $\mu$g. Groups of mice were sacrificed and tissues excised for counting at 4, 24 and 48 h after injection. In this preliminary study only blood and tumour were examined. The choice of blood as an index of normal tissue radioactivity was made since the concentration of radiolabelled fragment in this tissue had been shown to be higher than in any other normal tissue except kidney (Harwood *et al.*, 1985). Moreover, the presence of radioactivity in the blood is likely to be the dose limiting factor which could induce toxicity to the bone marrow and cause myelosuppression. The results were calculated as the absolute concentration ($\mu g\, g^{-1}$) of F(ab′)2-1C12.

The results at the 4 $\mu$g dose level (Figure 1a) show that by 4 h after injection the tumour accumulates approximately 0.15 $\mu$g F(ab′)2-1C12 $g^{-1}$ which is maintained up to 24 h and then

Correspondence: G.T. Rogers
Received 12 February 1986; and in revised form, 1 April 1986.

© The Macmillan Press Ltd., 1986

**Figure 1a–d** Absolute mean uptake of F(ab')2-1C12 into tumour (O——O) and blood (●——●) for administered doses ranging from 4–380μg per mouse over the time period 4–48 h after injection. The limiters represent the s.e. variation for each group of mice.

falls to 0.09 $\mu g\, g^{-1}$ by 48 h. Concentrations of fragment in the blood were initially much higher than that in tumour but fell rapidly to about 0.04 $\mu g\, g^{-1}$ at 24 h and were almost undetectable by 48 h. These results are in accordance with our previous findings with F(ab')2-1C12 where 4.0% of the injected dose was present $g^{-1}$ tumour up to 24 h after which time a net loss of fragment occurred (Harwood *et al.*, 1985). The rapid clearance of the fragment from the circulation was reflected in high tumour to blood ratios at 48 h (Table I). This phenomenon with fragments has been well established by many laboratories (Wilbanks *et al.*, 1981; Buchegger *et al.*, 1983; Wahl *et al.*, 1983; Harwood *et al.*, 1985).

The effect of increasing the administered dose of F(ab')2-1C12 on the relative concentrations in tumour and blood are shown in Figures 1b–1d. As expected an increase in dose resulted in an increase in the absolute concentration of label in the tumour at each time point but the percentage of the injected dose associated with the tumour declined. Therefore, the higher the dose the more gradual the increase in uptake in the tumour.

It is also noteworthy that at all dose levels studied, circulatory clearance was incomplete at 24 h but essentially complete by 48 h. However, concentrations of label in the tumour, although diminished, were still appreciable at 48 h after injection. Table I summarises the effect of this on the tumour to blood ratios which were dose dependent at 24 h and decreased with increasing dose, but with the exception of the highest dose, were unaffected by dose changes at 48 h. These results stress the potential importance of circulatory clearance mechanisms in tumour therapy and have been discussed in the previous report (Rogers *et al.*, 1986).

The ratio (r) (see Table I) has been used to denote the increased uptake of fragment in tumour

**Table I** Relationship between the administered dose of F(ab')2-1C12 and the amount present in tumour at 4, 24 and 48 h after injection.

| *Time* | *Dose (μg)* | *4* | *58* | *117* | *234* | *380* |
|---|---|---|---|---|---|---|
| 4 | T:B | 0.34 | 0.52 | – | 0.35 | 0.32 |
| | % inj. dose | 4.0 | 1.53 | — | 1.20 | 1.20 |
| | Uptake (μg) | 0.16 | 0.89 | — | 2.80 | 4.50 |
| | Ratio (r) | 1.00 | 5.50 | — | 17.5 | 28.0 |
| 24 | T:B | 4.40 | 4.20 | 2.80 | 1.90 | 1.70 |
| | % inj. dose | 4.10 | 1.50 | 0.96 | 0.70 | 0.65 |
| | Uptake (μg) | 0.16 | 0.87 | 1.12 | 1.64 | 2.47 |
| | Ratio (r) | 1.00 | 5.40 | 7.0 | 10.2 | 15.4 |
| 48 | T:B | 13.0 | 15.3 | — | 13.0 | 6.2 |
| | % inj. dose | 2.20 | 1.10 | — | 0.60 | 0.50 |
| | Uptake (μg) | 0.09 | 0.64 | — | 1.40 | 1.90 |
| | Ratio (r) | 1.00 | 7.0 | — | 15.6 | 21.1 |

Results are shown as the percentage of the injected dose, the absolute 'uptake' in $\mu g\,g^{-1}$ and as the ratio (r). The ratio (r) denotes the increased 'uptake' as a factor over that achieved at the 4 μg dose level and was calculated as

$$(r) = \frac{\text{absolute 'uptake' for increased dose}}{\text{absolute 'uptake' for the } 4\,\mu g \text{ dose}}$$

T:B = tumour to blood ratio. The figures represent mean values for each group of mice. Variation between mice, ommitted for clarity, was within 12%.

with increasing doses as a factor over the uptake achieved at the 4 μg dose. Thus at 24 and 48 h after injection a 100-fold increase in administered fragment resulted in a 15- and 21-fold increase in concentration in the tumour respectively.

Although results at individual time points are useful the data becomes more meaningful as a guide to potential therapy if considered as a cumulative effect over a period. We have attempted to do this for the period 4–48 h using the data in Figures 1a–1d and calculated the areas under the

**Table II** Relative localisation of F(ab')2-1C12 in tumour compared to blood over the time period 4–48 h after injection.

| *Dose (μg)* | $A_b$ | $A_t$ | *Relative localisation* |
|---|---|---|---|
| 4 | 3.7 | 5.0 | 1.35 |
| 58 | 22 | 33 | 1.50 |
| 234 | 73 | 80 | 1.10 |
| 380 | 109 | 126 | 1.16 |

$A_b$ and $A_t$ represent the areas under the curves shown in Figures 1a–d for blood and tumour respectively. The relative localisation was calculated as $A_t/A_b$.

curves for tumour and blood (Table II). This shows that the increasing dose levels over the range studied do not significantly lower the relative localisation of F(ab')2-1C12 fragments in the tumour compared to their concentration in blood. It is worth noting that, since the concentration of fragment in tumour is still significant at the last time point studied but almost cleared from the blood, the effective localisation indicated by our data (see Table II) up to 48 h underestimates the actual effective localisation in practice. Experiments at time points beyond 48 h need to be carried out to confirm this.

Our experiments show that, despite the adverse effect of dose escalation on the tumour to blood ratios at 4 and 24 h, antibody fragments may be useful in tumour immunoradiotherapy. Our data indicate that increased doses of F(ab')2 fragments in man may help compensate for their reduced concentration at the tumour site with minimal effect on the normal tissue distribution.

The authors are grateful to Mr. G.A. Rawlins for originating the monoclonal antibody 1C12 and to the Cancer Research Campaign for support.

### References

BEGENT, R.H.J. (1985). Recent advances in tumour imaging: Use of radiolabelled anti-tumour antibodies. *Biochim. Biophys. Acta*, **780**, 151.

BEGENT, R.H.J., BAGSHAWE, K.D., PEDLEY, R.B. & 7 others. (1986). Use of second antibody in radioimmunotherapy. *Cancer Treatment Symposia* (in press).

BUCHEGGER, F., HASKELL, C.M., SCHREYER, M., SCAZZIGA, B.R., RANDIN, S., CARREL, S. & MACH, J.-P. (1983). Radiolabelled fragments of monoclonal antibodies against CEA for localisation of human colon carcinoma grated into nude mice. *J. Exp. Med.*, **158**, 413.

HARWOOD, P.J., BODEN, J., PEDLEY, R.B., RAWLINS, G., ROGERS, G.T. & BAGSHAWE, K.D. (1985). Comparative tumour localisation of antibody fragments and intact IgG in nude mice bearing a CEA-producing human colon tumour xenograft. *Eur. J. Cancer Clin. Oncol.*, **21**, 1515.

LAMOYLE, E. & NISONOFF, A. (1983). Preparation of F(ab')2 fragments from mouse IgG of various subclasses. *J. Immunol. Meth.*, **56**, 235.

LARSON, S.M., CARRASQUILLO, J.A. & REYNOLDS, J.C. (1984). Radioimmunodetection and radioimmunotherapy. *Cancer Invest.*, **2**, 363.

MACH, J.-P., CARREL, S., FORNI, M., RITSCHARD, J., DONATH, A. & ALBERTO, P. (1981). Tumour localisation of radiolabelled antibodies against carcino-embryonic antigen in patients with carcinoma. *N. Engl. J. Med.*, **303**, 5.

ROGERS, G.T., HARWOOD, P.J., PEDLEY, R.B., BODEN, J., RAWLINS, G. & BAGSHAWE, K.D. (1985). Dynamics of monoclonal antibody distribution and prolonged tumour localisation in nude mice bearing a human CEA-producing colon carcinoma xenograft. *Tumour Biol.*, **6**, 453.

ROGERS, G.T., BODEN, J., HARWOOD, P.J., PEDLEY, R.B., RAWLINS, G.A. & BAGSHAWE, K.D. (1986). Dose-dependent localisation of a monoclonal F(ab')2 fragment against CEA in the mouse xenograft model. *Eur. J. Cancer Clin. Oncol.*, **22**, 709.

WAHL, R.L., PARKER, C.W. & PHILPOTT, G.W. (1983). Improved radioimaging and tumour localisation with monoclonal F(ab')2. J. Nucl. Med., **24**, 316.

WILBANKS, T., PETERSON, J.A., MILLER, S., KAUFMAN, L., ORTENDAHL, D. & CERIANI, R.L. (1981). Localisation of mammary tumours *in-vivo* with I-labelled Fab fragments of antibodies against mouse mammary epithelial (MME) antigens. *Cancer*, **48**, 1768.

ZALCBERG, J.R., THOMPSON, C.H., LICHTENSTEIN, M. & McKENZIE, F.C. (1984). Tumour immunotherapy in the mouse with use of $^{131}$I-labelled monoclonal antibodies. *J. Natl Cancer Inst.*, **72**, 697.